# Evaluation of clinical prediction models (part 1): from development to external validation

**DOI:** 10.1136/bmj-2023-074819

**Published:** 2024-01-08

**Authors:** Gary S Collins, Paula Dhiman, Jie Ma, Michael M Schlussel, Lucinda Archer, Ben Van Calster, Frank E Harrell, Glen P Martin, Karel G M Moons, Maarten van Smeden, Matthew Sperrin, Garrett S Bullock, Richard D Riley

**Affiliations:** 1 Centre for Statistics in Medicine, Nuffield Department of Orthopaedics, Rheumatology and Musculoskeletal Sciences, University of Oxford, Oxford OX3 7LD, UK; 2 Institute of Applied Health Research, College of Medical and Dental Sciences, University of Birmingham, Birmingham, UK; 3 National Institute for Health and Care Research (NIHR) Birmingham Biomedical Research Centre, UK; 4 KU Leuven, Department of Development and Regeneration, Leuven, Belgium; 5 Department of Biomedical Data Sciences, Leiden University Medical Centre, Leiden, Netherlands; 6 EPI-Centre, KU Leuven, Belgium; 7 Department of Biostatistics, Vanderbilt University, Nashville, TN, USA; 8 Division of Informatics, Imaging and Data Science, Faculty of Biology, Medicine and Health, University of Manchester, Manchester Academic Health Science Centre, Manchester, UK; 9 Julius Centre for Health Sciences and Primary Care, University Medical Centre Utrecht, Utrecht University, Utrecht, Netherlands; 10 Department of Orthopaedic Surgery, Wake Forest School of Medicine, Winston-Salem, NC, USA; 11 Centre for Sport, Exercise and Osteoarthritis Research Versus Arthritis, University of Oxford, Oxford, UK

## Abstract

Evaluating the performance of a clinical prediction model is crucial to establish its predictive accuracy in the populations and settings intended for use. In this article, the first in a three part series, Collins and colleagues describe the importance of a meaningful evaluation using internal, internal-external, and external validation, as well as exploring heterogeneity, fairness, and generalisability in model performance.

Healthcare decisions for individuals are routinely made on the basis of risk or probability.^1^ Whether this probability is that a specific outcome or disease is present (diagnostic) or that a specific outcome will occur in the future (prognostic), it is important to know how these probabilities are estimated and whether they are accurate. Clinical prediction models estimate outcome risk for an individual conditional on their characteristics of multiple predictors (eg, age, family history, symptoms, blood pressure). Examples include the ISARIC (International Severe Acute Respiratory and Emerging Infection Consortium) 4C model for estimating the risk of clinical deterioration in individuals with acute COVID-19,^2^ or the PREDICT model for estimating the overall and breast cancer specific survival probability at five years for women with early breast cancer.^3^ Clinical decision making can also be informed by models that estimate continuous outcome values, such as fat mass in children and adolescents, although we focus on risk estimates in this article.^4^ With increasing availability of data, pressures to publish, and a surge in interest in approaches based on artificial intelligence and machine learning (such as deep learning and random forests^5^ ^6^), prediction models are being developed at high volume. For example, diagnosis of chronic obstructive pulmonary disease has >400 models,^7^ cardiovascular disease prediction has >300 models,^8^ and covid-19 has >600 prognostic models.^9^

Despite the increasing number of models, very few are routinely used in clinical practice owing to issues including study design and analysis concerns (eg, small sample size, overfitting), incomplete reporting (leading to difficulty in fully appraising prediction model studies), and no clear link into clinical decision making. Fundamentally, there is often an absence or failure to fairly and meaningfully evaluate the predictive performance of a model in representative target populations and clinical settings. Lack of transparent and meaningful evaluation obfuscates judgments about the potential usefulness of the model, and whether it is ready for next stage of evaluation (eg, an intervention, or cost effectiveness study) or requires updating (eg, recalibration). To manage this deficit, this three part series outlines the importance of model evaluation and how to undertake it well, to help researchers provide a reliable and fair picture of a model’s predictive accuracy.

In this first article, we explain the rationale for model evaluation, and emphasise that it involves examining a model’s predictive performance at multiple stages, including at model development (internal validation) and in new data (external validation). Subsequent papers in this series consider the study design and performance measures used to evaluate the predictive accuracy of a model (part 2^10^) and the sample size requirements for external validation (part 3^11^). Box 1 provides a glossary of key terms.

Box 1 **Glossary of terms**

**Calibration**

Agreement between the observed outcomes and estimated risks from the model. Calibration should be assessed visually with a plot of the estimated risks on the x axis and the observed outcome on the y axis with smoothed flexible calibration curve in the individual data. Calibration can also be quantified numerically with the calibration slope (ideal value 1) and calibration-in-the-large (ideal value 0).

**Calibration-in-the-large**

Assesses mean (overall) calibration and quantifies any systematic overestimation or underestimation of risk, by comparing the mean number of predicted outcomes and the mean number of observed outcomes.

**Calibration slope**

Quantifies the spread of the estimated risks from the model relative to the observed outcomes. A slope <1 suggests that the spread of estimated risks are too extreme (ie, too high for individuals at high risk, and too low for those at low risk). Slope >1 suggests that the spread of estimated risks are too narrow.

**Discrimination**

Assesses how well the predictions from the model differentiate between those with and without the outcome. Discrimination is typically quantified by the c statistic (sometimes referred to as the AUC or AUROC) for binary outcomes, and the c index for time-to-event outcomes. A value of 0.5 indicates that the model is not better than a coin toss, and a value of 1 denotes perfect discrimination (ie, all individuals with the outcome have higher estimated risks than all individuals without the outcome). What defines a good c statistic value is context specific.

**Overfitting**

When the prediction model fits unimportant idiosyncrasies in the development data, to the point that the model performs poorly in new data, typically with miscalibration reflected by calibration slopes less than 1.

**Parameter tuning**

Finding the best settings for a particular model building strategy.

**Shrinkage**

Counteracting against overfitting by deliberately inducing bias in the predictor effects by shrinking them towards zero

AUC=area under the curve; AUROC=area under the receiver operating characteristic curve.

**Summary points**

Clinical prediction models use a combination of variables to estimate outcome risk for individuals
Evaluating the performance of a prediction model is critically important and validation studies are essential, as a poorly developed model could be harmful or exacerbate disparities in either provision of health care or subsequent healthcare outcomes
Evaluating model performance should be carried out in datasets that are representative of the intended target populations for the model’s implementation
A model’s predictive performance will often appear to be excellent in the development dataset but be much lower when evaluated in a separate dataset, even from the same population
Splitting data at the moment of model development should generally be avoided as it discards data leading to a more unreliable model, whilst leaving too few data to reliably evaluate its performance
Concerted efforts should be made to exploit all available data to build the best possible model, with better use of resampling methods for internal validation, and internal-external validation to evaluate model performance and generalisability across clusters

## Why do we need to evaluate prediction models?

During model development (or training), study design and data analysis aspects will have an impact on the predictive performance of the model in new data from some target population. A model’s predictive performance will often appear excellent in the development dataset but be much lower when evaluated in a separate dataset, even from the same population, often rendering the model much less accurate. The downstream effect is that the model will be less useful and even potentially harmful, including exacerbating inequalities in either provision of healthcare or subsequent healthcare outcomes. Therefore, once a prediction model has been developed, it is clearly important to carry out a meaningful evaluation of how well it performs.

Evaluating the performance of a prediction model is generally referred to as validation.^12^ However, the term validation is ill defined, used inconsistently,^13^ and evokes a sense of achieving some pre-defined level of statistical or clinical usefulness. A validated model might even (albeit wrongly) be considered a sign of approval for use in clinical practice. Many prediction models that have undergone some form of validation will still have poor performance, either a substantial decrease in model discrimination or, more likely, in calibration (see box 1 for definitions of these measures, and part 2 of our series for more detailed explanation^10^). Yet determining what level of predictive performance is inadequate (eg, how miscalibrated a model needs to be to conclude poor performance) is subjective. Many validation studies are also too small, a consideration that is frequently overlooked, leading to imprecise estimation of a model’s performance (see part 3 on guidance for sample size^11^). Therefore, referring to a model as having been “validated” or being “valid,” just because a study labelled as validation has been conducted, is unhelpful and arguably misleading. Indeed, variation in performance over different target populations,^14^ or different time periods and places (eg, different centres or countries), is to be expected^15^ and so a model can never be proven to be always valid (nor should we expect it to be^16^).

**Fig 1:**
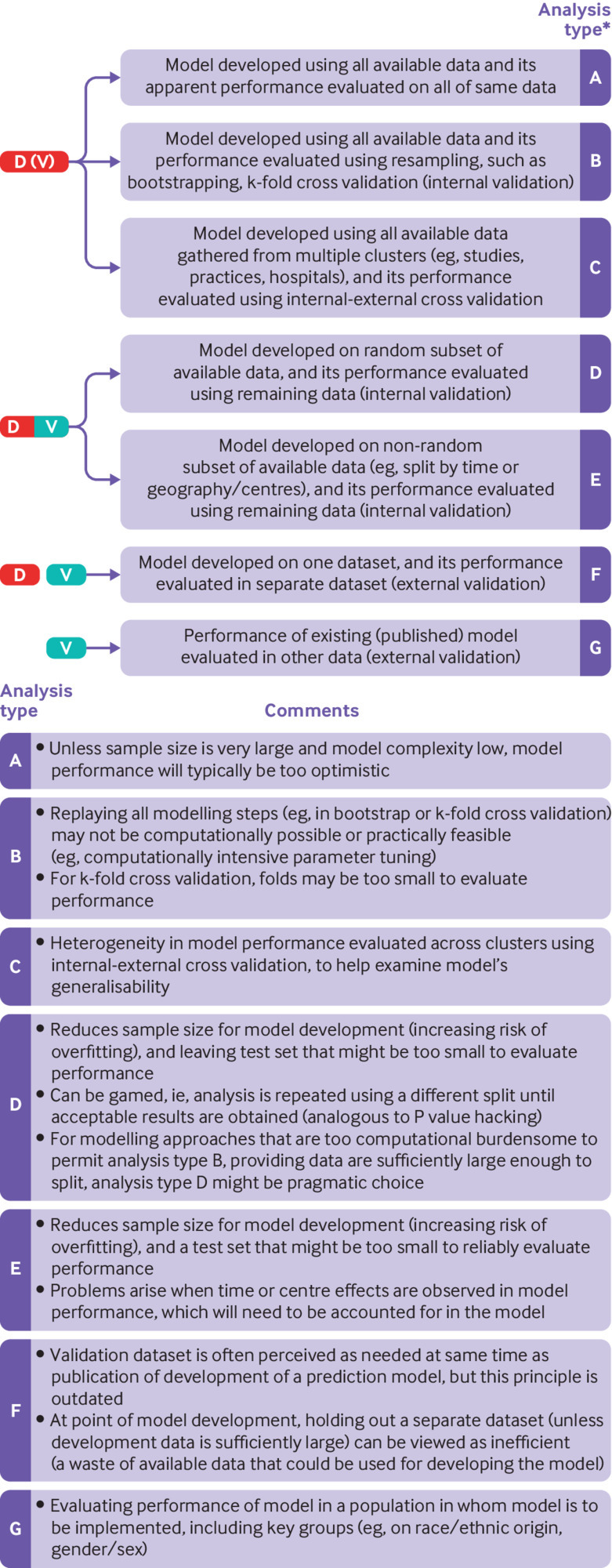
Different study design and approaches to develop and evaluate the performance of a multivariable prediction model (D=development; V=validation (evaluation)). Adapted from Collins GS, Reitsma JB, Altman DG, Moons KGM. Transparent reporting of a multivariable prediction model for individual prognosis or diagnosis (TRIPOD): the TRIPOD statement. *BMJ* 2015;350:g7594.^17^ *A study can include more than one analysis type

Figure 1 shows a summary of the different study designs and approaches involving prediction model development and validation. The decision of which validation to carry out depends on the research question that is being asked and the availability of existing data. Regardless of the development approach, the validation component is essential, because any study developing a new prediction model should, without exception, always evaluate the model’s predictive performance for the target population, setting and outcome of interest. We now outline the various options for model evaluation, moving from internal validation to external validation.

## Evaluation at model development: internal validation approaches

At the stage of model development, depending on the availability, structure (eg, multiple datasets, multicentre) and size of the available data, investigators are faced with deciding how best to use the available data to both develop a clinical prediction model and evaluate its performance in an unbiased, fair, and informative manner. When the evaluation uses the same data (or data source) as used for model development, the process is referred to as internal validation. For example, the Transparent Reporting of a multivariable prediction model for Individual Prognosis Or Diagnosis (TRIPOD) reporting guideline requires users to “specify type of model, all model-building procedures (including any predictor selection), and method for internal validation.”^17^ ^18^

Widely used approaches for internal validation are based on data splitting (using a subset of the data for development and the remainder for evaluation) or resampling (eg, k-fold cross validation or bootstrapping; table 1). For very large datasets, and computationally intensive model building procedures (eg, including parameter tuning; box 1), the decision on which approach is used for internal validation could be a pragmatic one. Nevertheless, some approaches are inefficient and uninformative, and, especially in small sample sizes, might even lead to biased, imprecise and optimistic results and ultimately misleading conclusions. Therefore, we now describe the advantages and disadvantages of several strategies in detail.

**Table 1:** Different approaches for evaluating model performance

Type of validation	Description	Comments
Apparent performance	Performance of the model when evaluated in the same data used to develop the model.	When the sample is of small to moderate size (see part 3 in this series^11^), the apparent performance will be optimistic (upwardly biased). As the sample size increases, the optimism will decrease. For very large sample sizes, there will be no discernible optimism, and apparent performance will be unbiased.
Internal validation	Estimating model performance for the underlying population used to develop the model.	A minimal expectation, and one of the TRIPOD statement reporting recommendations (item 10b), is that studies developing a prediction model should carry out an internal validation of that model in the population in whom it is intended to be used. Common internal validation approaches include data splitting, and variations of k-fold cross validation and bootstrapping.
Split sample validation	Data are (usually randomly) split into two: one used to develop the model, one used to evaluate the performance of the model.	Split sample validation is generally advised against. When the available data are small to moderate, splitting data will create a dataset that is insufficient for model development (increasing the likelihood of overfitting), and a dataset that is insufficient to evaluate the performance of the model. Conversely when the sample size is large, there is little risk of overfitting, and thus no new information is gained in evaluating the model in the validation data. Randomly splitting the dataset also opens up the danger of multiple looks until satisfactory results are obtained.
k-fold cross validation	Model performance is evaluated by splitting the data into k groups, where k-1 groups are used to develop a (temporary) model (repeating the model building steps used to develop the model on all the data) and the group left out is used to evaluate the performance of the temporary model. This process is repeated k times, each time leaving out a different group, producing k values of each performance measure. The performance of the developed model is then taken as the average (or median) over the k performance measures.	All the available data are used to develop the model and performance of this model is then evaluated using k-fold cross validation (or repeat k-fold cross validation) and bootstrapping to get an unbiased or least unbiased estimate of model performance in the underlying population in whom the model is intended. The complexity of implementing either k-fold cross validation or bootstrapping increases when both missing data and selection of non-linear terms (eg, using restricted cubic splines or fractional polynomials) are part of the model building process.
Bootstrapping	Bootstrapping is a resampling technique, where a bootstrap sample is created by randomly sampling (with replacement) from the original data. In the enhanced bootstrap, a model is developed (repeating the model building steps used to develop the model on all the data) in each bootstrap sample and its performance evaluated in this sample as well as the original dataset to get an estimate of optimism of model performance. This process is repeated many times and the average optimism calculated, which is then subtracted from the apparent performance.	
Internal-external cross validation	Heterogeneity in performance of the model across clusters. A cluster could be a dataset (when multiple datasets are available, eg, from an IPDMA) or centre (eg, hospitals, general practices). Similar to k-fold cross validation, all clusters with one omitted are used to develop a model, and its performance evaluated on the omitted cluster. This process is repeated taking out a different cluster, so that each cluster is omitted once from the development and used as a test dataset.	All available data are used to develop the model and IECV is used to examine heterogeneity in model performance. IECV can also be used to explore clusters where model performance is poor (and explore reasons), which could lead to dropping the cluster from the data and a new model developed.
External validation	Estimating model performance in a different sample of data to that used to develop the model. The data might be the from same (or similar to) the population or setting used for model development (assessing reproducibility), or might be from a different population or setting (assessing transportability). Another type of validation is where researchers evaluate model performance across multiple populations and settings, where each is relevant to the intended use (assessing generalisability).^14^	External validation at the model development stage is not an efficient use of available data and should not be carried out solely to meet over-zealous and misinformed editorial or reviewer requirements. External validation should be used to evaluate model performance in subsequent studies in new data that are representative of a target population. Using existing data that are merely conveniently available provide limited, and often misleading, information on model performance. External validation studies could also be used to evaluate model performance in settings that are intentionally different (eg, a model developed for adults, but subsequently in a different study evaluated in children^19^), or to explore the model performance when the predictor or outcome definitions (including time horizon) are different (eg, a model to predict an outcome at one year, but evaluated for a two year outcome).
Temporal validation	Evaluating the performance of an existing prediction model in data from the same or similar setting in a different time period.	At model development, temporal validation is rarely useful and should be avoided. However, understanding whether model performance is changing (and importantly deteriorating) over the study period is useful to understand and ideally rectify.
Geographical or spatial validation	Evaluating the performance of an existing prediction model in data collected from an appropriate population in different centres (to the model development).	At model development, geographical validation is rarely useful, particularly when all the data can be used to develop the model and heterogeneity in model performance across different centres can be explore using the IECV approach. If data are particularly large, and analysis computationally burdensome, then leaving out a cluster (eg, a centre or country) is a pragmatic compromise that can be considered.

IECV=internal-external cross validation; IPDMA=individual participant data meta-analysis.

### Apparent performance

The simplest approach is to use all the available data to develop a prediction model and then directly evaluate its performance in exactly the same data (often referred to as apparent performance). Clearly, using this approach is problematic, particularly when model complexity and the number of predictors (model parameters to be estimated) is large relative to the number of events in the dataset (indicative of overfitting).^20^ The apparent performance of the model will therefore typically be optimistic; that is, when the model is subsequently evaluated in new data, even in the same population, the performance will usually be much lower. For small datasets, the optimism and uncertainty in the apparent performance can be substantial. As the sample size of the data used to develop the model increases, the optimism and uncertainty in apparent performance will decrease, but in most healthcare research datasets some (non-negligible) optimism will occur.^20^ ^21^

**Fig 2:**
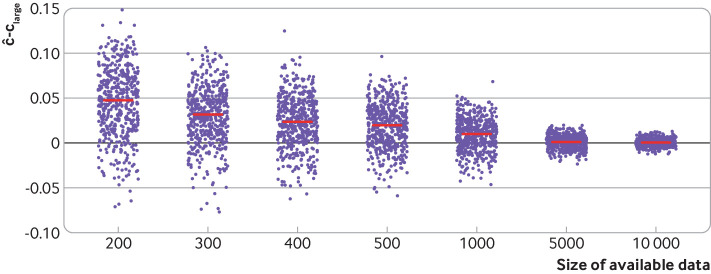
Variability and overestimation of apparent performance compared to large sample performance, for a model to predict in-hospital mortality within 28 days of trauma injury with increasing sample size of the model development study. ĉ denotes the apparent performance estimate and c_large_ denotes the performance of the model in the entire CRASH-2 population (n=20 207).^22^ Red lines=mean ĉ−c_large_ for each sample size. Jitter has been added to aid display. ĉ−c_large_=0 implies no systematic overestimation or underestimation of ĉ

To illustrate apparent performance, we consider a logistic regression model for predicting in-hospital mortality within 28 days of trauma injury in patients with an acute myocardial infarction using data from the CRASH-2 clinical trial (n=20 207, 3089 died within 28 days)^22^ using 14 predictors including four clinical predictors (age, sex, systolic blood pressure, and Glasgow coma score) and 10 noise predictors (ie, truly unrelated to the outcome). Varying the sample size between 200 and 10000, models are fit to 500 subsets of the datasets that are created by resampling (with replacement) from the entire CRASH-2 data and each model’s apparent performance calculated. For simplicity, we focus primarily on the c statistic, a measure of a prediction models discrimination (how well the model differentiates between those with and without the outcome, with a value of 0.5 denoting no discrimination and 1 denoting perfect discrimination; see box 1 and part 2 of the series^10^). Figure 2 shows the magnitude and variability of the difference in the c statistic for the apparent performance estimate compared with the large sample performance value of 0.815 (ie, a model developed on all the available data). For small sample sizes, there is a substantial difference (estimates are systematically much larger) and large variation, with the apparent c statistic ranging anywhere from 0.7 to just under 1. This variability in apparent performance decreases as the sample size increases, and for very large sample sizes, the optimism in apparent performance is negligible and thus a good estimate of the underlying performance in the full (CRASH-2) population.

### Random split

Randomly splitting a dataset is often erroneously perceived as a methodological strength—it is not. Authors also often label the two datasets (created by splitting) as independent; despite no overlap in patients, the label “independent” is a misnomer, because they clearly both come from the same dataset (and data source).

**Fig 3:**
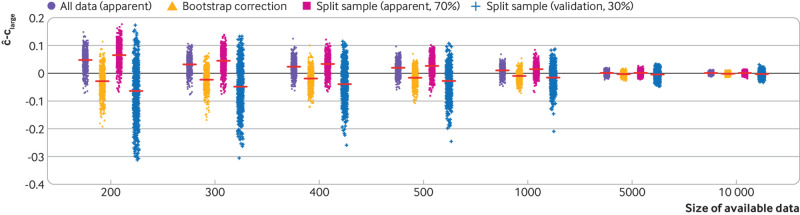
Variability and overestimation of the apparent and internal (split sample and bootstrap) validation performance compared with the large sample performance, for a model to predict in-hospital mortality within 28 days of trauma injury with increasing sample size of the model development study. ĉ denotes the apparent performance estimate and c_large_ denotes the performance of the model in the entire CRASH-2 population (n=20 207). The red lines denote the mean ĉ−c_large_ for each sample size and for each approach. Jitter has been added to aid display. Split sample (apparent, 70%)=70% of the available data were used to develop the model, and its (apparent) performance evaluated in this same data. Split sample (validation, 30%)=the performance of the model (developed in 70% of the available data) in the remaining 30% of the data. ĉ−c_large_=0 implies no systematic overestimation or underestimation of ĉ

Randomly splitting obviously creates two smaller datasets,^23^ and often the full dataset is not even large enough to begin with. Having a dataset that is too small to develop the model increases the likelihood of overfitting and producing an unreliable model,^20^ ^21^ ^24^ ^25^ ^26^ and having a test set that is too small will not be able to reliably and precisely estimate model performance—this is a clear waste of precious information^27^ ^28^ ^29^ (see part 3 in this series^11^). Figure 3 illustrates the impact of sample size on performance (the c statistic) of a prediction model using a random split sample approach. Using the same approach as before, a logistic regression model for predicting 28 day mortality in patients with acute myocardial infarction was developed using 14 predictors (age, sex, systolic blood pressure, Glasgow coma score, and 10 noise predictors). The models are fit and evaluated in 500 split sample subsets of the CRASH-2 data, whereby 70% of observations are allocated to the development data and 30% to the test data (eg, for total sample size of n=200, 140 are used for development and 60 are used for evaluation). The results clearly show that for small datasets, using a split sample approach is inefficient and unhelpful. The apparent c statistic of the developed model is too large (ie, optimistic) compared with the large sample performance and noticeably variable, while the test set evaluation (validation) shows that the develop model’s c statistic is much lower and highly variable, and underestimated relative to the large sample performance of the model (again, indicative of overfitting during model development due to too few data). Also, when fewer participants (eg, 90:10 split) are assigned to the test set, even more variability is seen in the model’s observed test set performance (supplementary fig 1).

As sample size increases, the difference between the split sample apparent performance and the test set performance reduces. In very large sample sizes, the difference is negligible. Therefore, data splitting is unnecessary and not an improvement on using all the data for model development and reporting apparent performance when the sample size is large or using internal validation methods (eg, bootstrapping, see below) when sample size is smaller. This observation is not new and has been stated in the methodological literature over 20 years ago,^30^ but the message has still not made it to the mainstream biomedical and machine learning literature.

For models with high complexity (eg, deep learners) that prohibit resampling of the full dataset (eg, using bootstrapping), a split sample approach might still be necessary. Similarly, sometimes two or more datasets could be available (eg, from two e-health databases) but not combinable, owing to local restrictions on data sharing, such that a split sample is enforced. In these situations, we strongly recommended having very large development and test datasets, as otherwise the developed model might be unstable and test performance unreliable, rendering the process futile. Concerns of small sample sizes can be revealed by instability plots and measures of uncertainty.^31^

In addition to the issues of inefficiency and increased variability (instability), randomly splitting the dataset also opens up the danger of multiple looks and spin. That is, if poor performance is observed when evaluating the model in the test portion of the randomly split dataset, researchers could be tempted to repeat the analysis, splitting the data again until the desired results are obtained, similar to P hacking, and thus misleading readers into believing the model has good performance.

### Resampling approaches: bootstrapping and k-fold cross validation

Unlike the split sample approach, which evaluates a specific model, bootstrapping evaluates the model building process itself (eg, predictor selection, imputation, estimation of regression coefficients), and estimates the amount of optimism (due to model overfitting) expected when using that process with the sample size available.^32^ This estimate of optimism is then used to produce stable and approximately unbiased estimates of future model performance (eg, c statistic, calibration slope) in the population represented by the development dataset.^30^ The process starts with using the entire dataset to develop the prediction model and its apparent performance estimated. Bootstrapping is then used to estimate and adjust for optimism, in both the estimates of model performance and the regression coefficients (box 2).

Box 2 **Using bootstrapping for internal validation**

The steps to calculate optimism corrected performance using bootstrapping are:

1. Develop the prediction model using the entire original data and calculate the apparent performance.
2. Generate a bootstrap sample (of the same size as the original data), by sampling individuals with replacement from the original data.
3. Develop a bootstrap model using the bootstrap sample (applying all the same modelling and predictor selection methods, as in step 1):
  - Determine the apparent performance (eg, c statistic, calibration slope) of this model on the bootstrap sample (bootstrap performance).
  - Determine the performance of the bootstrap model in the original data (test performance).
4. Calculate the optimism as the difference between the bootstrap performance and the test performance.
5. Repeat steps 2 to 4 many times (eg, 500 times).
6. Average the estimates of optimism in step 5.
7. Subtract the average optimism (from step 6) from the apparent performance obtained in step 1 to obtain an optimism corrected estimate of performance.

The variability in the optimism corrected estimates, across the bootstrap samples, can also be reported to demonstrate stability.^33^ The bootstrap models produced in step 2 will vary (and differ from the prediction model developed on the entire data), but these bootstrap models are only used in the evaluation of performance and not for individual risk prediction. Steyerberg and colleagues have shown that the expected optimism could precisely be estimated with as few as 200 bootstraps with minor sampling variability; with modern computational power, we generally recommend at least 500 bootstraps.^34^ An additional benefit of this bootstrap process is that the value of optimism corrected calibration slope can be used to adjust the model from any overfitting by applying it as shrinkage factor to the original regression coefficients (predictor effects).^32^ ^35^ ^36^

Figure 3 shows that using all the available data to develop a model and using bootstrapping to obtain an estimate of the model’s optimism corrected performance, is an efficient approach to internal validation, leading to estimates of model performance that are closest to the large sample performance (eg, compared to a split sample approach), as shown elsewhere^30^ (supplementary table 1). For very large datasets, the computational burden to carry out bootstrapping can prohibit its use; in these instances, however, little is achieved over using the entire dataset to both derive and evaluate a model, because the estimate of apparent performance should be a good approximation of the underlying large sample performance of the model.

Another resampling method, k-fold cross validation, will often perform comparably to bootstrapping.^30^ Like bootstrapping, all available data are used to develop the model, and all available data are used to evaluate model performance. k-fold cross validation can be seen an extension of the split sample approach but with a reduction in the bias and variability in estimation of model performance (box 3).

Box 3 **Use of k-fold cross validation for internal validation**

**Fig 4:**
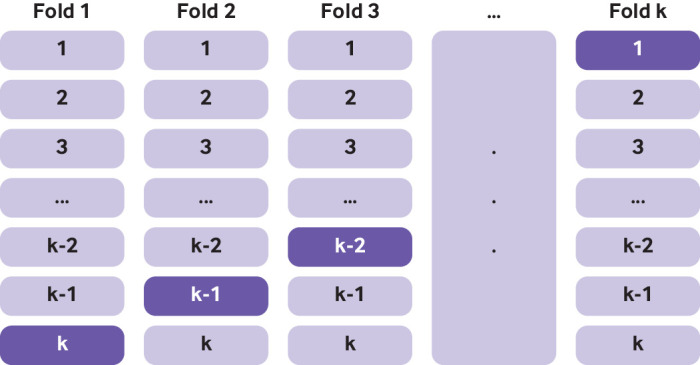
Graphical illustration of k-fold cross validation. Non-shaded parts used for model development; shaded part used for testing

The process of k-fold cross validation entails splitting the data into “k” equal sized groups. A model is developed in k-1 groups, and its performance (eg, c statistic) evaluated in the remaining group. This process is carried out k times, so that each time a different set of k-1 groups is used to develop the model and a different group is used to evaluate model performance (fig 4). The average performance over the k iterations is taken as an estimate of the model performance.

In practice, the value of k is usually taken to be 5 or 10; cherry picking k should be avoided. Repeated k-fold cross validation (where k-fold validation is repeated multiple times and results averaged across them) will generally improve on k-fold cross validation.

### Non-random split (at model development)

Alternative splitting approaches include splitting by time (referred to as temporal validation) or by location (referred to as geographical or spatial validation).^37^ However, they remove the opportunity to explore and capture time and location features during model development to help explain variability in outcomes.

In a temporal validation, data from one time period are used to develop the prediction model while data from a different (non-overlapping) time period are used to evaluate its performance. The concern, though, is selecting which time period should be used to develop the model, and which to use for evaluation. Using data from the older time period for model development might not reflect current patient characteristics (predictors and outcomes) or current care. Conversely, using the more contemporary time period to develop the model leaves the data from an older time period to evaluate the performance, and so only provides information on the predictive accuracy in a historical cohort of patients. Neither option is satisfactory, and this approach (at the moment of model development) is not recommended. For example, improvements over time in surgical techniques have led to larger number of patients surviving surgery,^38^ and thus the occurrence of the outcome being predicted will decrease over time, which will have an impact on model calibration. Methods such as continual (model) updating should therefore be considered to prevent calibration drift or dynamic prediction models.^39^ Temporal recalibration is another option^40^ where the predictor effects are estimated in the whole dataset, but the baseline risk is estimated in the most recent time window.

In a geographical or spatial validation, data from one geographical location (or hospitals, centres) are used to develop the model, while data from a separate geographical location are used to evaluate the model. As with other data splitting approaches previously discussed, in most (if not all) instances, there is often little to be gained in splitting, and rather a missed opportunity in using all available data to develop a model with wider generalisability. However, if data from many geographical regions (or centres) are available to develop a model, comprising a very large number of observations (and outcomes), and computational burden of model development prohibits k-fold cross validation or bootstrapping, leaving out one or more regions or centres to evaluate performance might not be too detrimental.^41^ As with the random split approach, researchers might be tempted to split the data (eg, into different time periods and lengths, different centres) repeatedly until satisfactory performance has been achieved—this approach should be avoided. If splitting is to be considered, the splits should be done only once (ie, no repeated splitting until good results are achieved), ensuring that the sample sizes for development and evaluation are of sufficient size.

## Evaluation at model development: internal-external cross validation

Data from large electronic health record databases, multicentre studies, or individual participant data from multiple studies are increasingly being made available and used for prediction model purposes.^15^ ^42^ Researchers might be tempted to perform some form of (geographical or spatial) splitting, whereby only a portion (eg, a group of centres, regions of a country, or a group of studies) is used to develop the model, and the remaining data is used to evaluate its performance. However, internal-external cross validation is a more efficient and informative approach^43^ ^44^ ^45^ ^46^ that examines heterogeneity and generalisability in model performance (box 4).

Box 4 **Internal-external cross validation**

**Fig 5:**
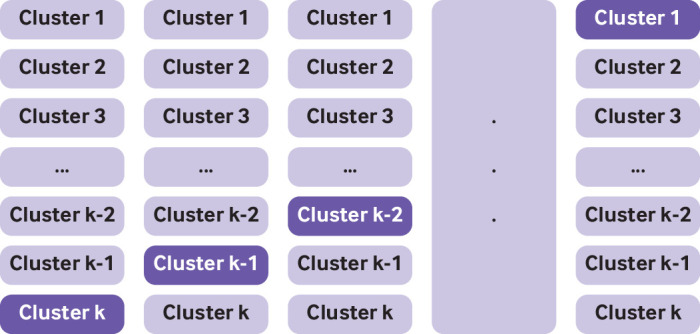
Graphical illustration of internal-external cross validation. Non-shaded parts used for model development; shaded part used for testing

Internal-external validation exploits a common feature present in many datasets, namely that of clustering (eg, by centre, geographical region, or study). Instead of partitioning the data into development and validation cohorts, all the data are used to build the prediction model and iteratively evaluate its performance. The performance of this model (developed on all the data) is then examined using cross validation by cluster, where a cluster is held out (eg, a centre, geographical region, study) and the same model building steps (as used on the entire data) are applied to the remaining clusters. The model is then evaluated in the held-out cluster (ie, estimates of calibration and discrimination along with confidence intervals). These steps are repeated, each time taking out a different cluster^44^ thereby allowing the generalisability and heterogeneity of performance to be examined across clusters (using meta-analysis techniques; fig 5).

The results can then be presented in a forest plot to aid interpretation, and a summary estimate calculated using (random effects) meta-analysis. TRIPOD (transparent reporting of a multivariable prediction model for individual prognosis or diagnosis)-Cluster provides recommendations for reporting prediction model studies that have accounted for clustering during validation, including the approach of internal-external cross validation.^47^ ^48^

**Fig 6:**
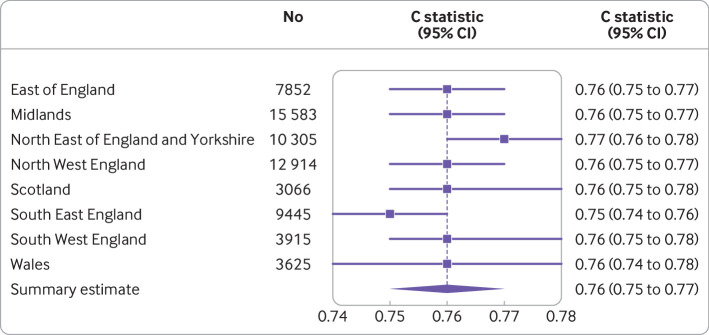
Internal-external cross validation of the ISARIC (International Severe Acute Respiratory and Emerging Infection Consortium) 4C model. Adapted from Gupta et al.^2^ Estimates and confidence intervals taken from original paper where they were reported to two decimal places.

For example, internal-external cross validation was used in the development of the ISARIC 4C model to identify individuals at increased risk of clinical deterioration in adults with acute covid-19.^2^ The authors used all their available data (n=74 944) from nine regions of the UK (each comprising between 3066 and 15 583 individuals) to develop the model but then, to examine generalisability and heterogeneity, performed an internal-external cross validation with eight regions in the model development and the ninth region held out for evaluation. The authors demonstrated that the model performed consistently across regions, with point estimates of the c statistic ranging from 0.75 to 0.77, and a pooled random effects meta-analysis estimate of 0.76 (95% confidence interval 0.75 to 0.77; fig 6).

## Evaluation using new data: external validation

External validation is the process of evaluating the performance of an existing model in a new dataset, differing to that used (and the source used) for model development. It is an important component in the pipeline of a prediction model, as its pursuit is to demonstrate generalisability and transportability of the model beyond the data (and population) used to develop the model (eg, in different hospitals, different countries).^49^ For example, Collins and Altman conducted an independent external validation of QRISK2 and the Framingham risk score (at the time recommended by National Institute for Health and Care Excellence in the UK), and demonstrated systematic miscalibration of Framingham, no net benefit at current (at the time) treatment thresholds, and the need for different treatment thresholds.^50^

Some journals refuse to publish model development studies without an external validation^51^; this stance is outdated and misinformed, and could encourage researchers to perform a meaningless and misleading external validation (eg, non-representative convenience sample, too small, even data splitting under the misnomer of external validation). Indeed, if the model development dataset is large and representative of the target population (including outcome and predictor measurement), and internal validation was done appropriately, then an immediate external validation might not even be needed.^14^ However, in many situations, the data used to develop a prediction model might not reflect the target population in whom the model is intended, and variation or lack of standardisation in measurements (including measurement error), poor statistical methods, inadequate sample size, handling of missing data (including missing important predictors), and changes in health care could all affect the model performance when applied to a target representative population.^52^ Supplementary figure 2 and supplementary table 2 demonstrates the impact of sample size in model development on performance at external validation. Thus, most prediction models need evaluation in new data to demonstrate where they should and should not be considered for deployment or further evaluation of clinical impact (eg, in a randomised clinical trial^53^).

External validations are needed because variations in healthcare provision, patient demographics, and local idiosyncrasies (eg, in outcome definitions) will naturally dictate the performance of a particular prediction model. Frameworks have been proposed to aid the interpretation of findings at external validation by examining the relatedness (eg, how similar in terms of case mix) of the external validation data to the development data, to explore (on a continuum) whether the validation assesses reproducibility (data are similar to the development data) or transportability (data are dissimilar to the development data).^54^ ^55^ The data used in an external validation study could be from the same population as used for model development, but at a different (more contemporary) time period, obtained subsequent to the model development.^56^ Indeed, continual or periodic assessment in the sample population is important to identify and deal with any model deterioration (eg, calibration drift^57^), which is expected owing to population or healthcare changes over time. However, researchers should also consider external validation in entirely different populations (eg, different centres or countries) or settings (eg, primary/secondary care or adults/children) where the model is sought to be deployed. External validation might even involve different definitions of predictors or outcome (eg, different prediction horizon) than used in the original development population.

External validation is sometimes included in studies developing a prediction model. However, as noted earlier, at the moment of model development, we generally recommend that all available data should be used to build the model, accompanied by a meaningful internal or internal-external cross validation. Using all the available data to develop a model implies that external validation studies should then (in most instances) be done subsequently and outside the model development study, each with a specific target population in mind (ie, each intended target population or setting for a given prediction model should have a corresponding validation exercise^14^). The more external validation studies showing good (or acceptable) performance, the more likely the model will also be useful in other untested settings—although clearly there is no guarantee.

Guidance on the design and analysis for external validation studies is provided in parts 2 and 3 of this series.^10^ ^11^ Despite the importance of carrying out an external validation, such studies are relatively sparse,^58^ and publication bias is most certainly a concern, with (generally) only favourable external validation studies published. Despite the rhetoric chanting for replication and validation, journals seem to have little appetite in publishing external validation studies (presumably and cynically with citations having a role), with preference for model development studies. It is not inconceivable that researchers (who developed the model) will be less likely to publish external validation studies showing poor or weak performance. Incentives for independent researchers to carry out an external validation are also a contributing factor—what are the benefits for them, with seemingly low appetite by journals to publish them, particularly when the findings are not exciting? Failure of authors to report or make the prediction model available will, either through poor reporting or for proprietary reasons,^59^ also be a clear barrier for independent evaluation, potentially leading to only favourable findings (by the model developers).

## Evaluation in subgroups: going beyond population performance to help examine fairness

Evaluating model performance typically focuses on measures of performance at the dataset level (eg, a single c statistic, or a single calibration plot or measure) as a proxy for the intended target population. While this performance is essential to quantify and report, concerted efforts should be made to explore potential heterogeneity and delve deeper into (generalisability of) model performance. Researchers should not only highlight where their model exhibits good performance, but also carry out and report findings from a deeper interrogation and identify instances, settings, and groups of people where the model has poorer predictive accuracy, because using such a model could have a downstream impact on decision making and patient care, and potentially harm patients. For example, in addition to exploring heterogeneity in performance across different centres or clusters (see above), researchers should be encouraged (indeed expected) to evaluate model performance in other key subgroups (such as sex/gender, race/ethnic group), as part of checking algorithmic fairness,^60^ especially when sample sizes are large enough, and when data have been collected in an appropriate way that represents the diverse range of people the model is intended to be used in.^61^ For example, in their external validation and comparison of QRISK2 and the Framingham risk score, Collins and Altman demonstrated miscalibration of the Framingham risk score, with systematic overprediction in men across all ages, and a small miscalibration of QRISK2 in those of older age.^50^

Introducing a new technology in clinical care, such as a prediction model, which is expected only to increase with the surge in interest and investment in artificial intelligence and machine learning, should ideally reduce but certainly not create or exacerbate any disparities in either provision of healthcare or indeed subsequent healthcare outcomes.^62^ ^63^ ^64^ Consideration of key subgroups is therefore important during the design (and data collection), analysis, reporting, and interpretation of findings.

## Conclusions

Evaluating the performance of a prediction model is critically important and therefore validation studies are essential. Here, we have described how to make the most of the available data to develop and, crucially, evaluate a prediction model from development to external validation. Splitting data at the moment of model development should generally be avoided because it discards data leading to a more unreliable model. Rather, concerted efforts should be made to exploit all available data to build the best possible model, with better use of resampling methods for internal validation, and internal-external validation to evaluate model performance and generalisability across clusters. External validation studies should be considered in subsequent research, preferably by independent investigators, to evaluate model performance in datasets that are representative of the intended target populations for the model’s implementation. The next paper in this series, part 2, explains how to conduct such studies.^10^

## Data Availability

The CRASH-2 and CRASH-3 data used in this paper are freely available at https://freebird.lshtm.ac.uk. The R code used to produce the figures and supplementary tables is available from https://github.com/gscollins1973/validationCRASH.

## Web extra

Extra material supplied by authors

Web appendix: Supplementary material

## Notes

Patient and public involvement: Patients or the public were not involved in the design, or conduct, or reporting, or dissemination of our research.

Provenance and peer review: Not commissioned, externally peer reviewed.

## References

[1] van Smeden M Reitsma JB Riley RD Collins GS Moons KG . Clinical prediction models: diagnosis versus prognosis. J Clin Epidemiol 2021;132:142-5. 10.1016/j.jclinepi.2021.01.009 33775387

[2] Gupta RK Harrison EM Ho A ISARIC4C Investigators . Development and validation of the ISARIC 4C Deterioration model for adults hospitalised with COVID-19: a prospective cohort study. Lancet Respir Med 2021;9:349-59. 10.1016/S2213-2600(20)30559-2 33444539 PMC7832571

[3] Wishart GC Azzato EM Greenberg DC . PREDICT: a new UK prognostic model that predicts survival following surgery for invasive breast cancer. Breast Cancer Res 2010;12:R1. 10.1186/bcr2464 20053270 PMC2880419

[4] Hudda MT Fewtrell MS Haroun D . Development and validation of a prediction model for fat mass in children and adolescents: meta-analysis using individual participant data. BMJ 2019;366:l4293. 10.1136/bmj.l4293 31340931 PMC6650932

[5] Christodoulou E Ma J Collins GS Steyerberg EW Verbakel JY Van Calster B . A systematic review shows no performance benefit of machine learning over logistic regression for clinical prediction models. J Clin Epidemiol 2019;110:12-22. 10.1016/j.jclinepi.2019.02.004 30763612

[6] Dhiman P Ma J Navarro CA . Reporting of prognostic clinical prediction models based on machine learning methods in oncology needs to be improved. J Clin Epidemiol 2021;138:60-72. 10.1016/j.jclinepi.2021.06.024 34214626 PMC8592577

[7] Bellou V Belbasis L Konstantinidis AK Tzoulaki I Evangelou E . Prognostic models for outcome prediction in patients with chronic obstructive pulmonary disease: systematic review and critical appraisal. BMJ 2019;367:l5358. 10.1136/bmj.l5358 31585960 PMC6776831

[8] Damen JAAG Hooft L Schuit E . Prediction models for cardiovascular disease risk in the general population: systematic review. BMJ 2016;353:i2416. 10.1136/bmj.i2416 27184143 PMC4868251

[9] Wynants L Van Calster B Collins GS . Prediction models for diagnosis and prognosis of covid-19: systematic review and critical appraisal. BMJ 2020;369:m1328. 10.1136/bmj.m1328 32265220 PMC7222643

[10] Riley RD Archer L Snell KIE . Evaluation of clinical prediction models (part 2): how to undertake an external validation study. BMJ 2023;383:e074820. 10.1136/bmj-2023-074820 PMC10788734 38224968

[11] Riley RD Snell KIE Archer L . Evaluation of clinical prediction models (part 3): calculating the sample size required for an external validation study. BMJ 2023;383:e074821. 10.1136/bmj-2023-074821 PMC11778934 38253388

[12] Justice AC Covinsky KE Berlin JA . Assessing the generalizability of prognostic information. Ann Intern Med 1999;130:515-24. 10.7326/0003-4819-130-6-199903160-00016 10075620

[13] Kim DW Jang HY Ko Y . Inconsistency in the use of the term “validation” in studies reporting the performance of deep learning algorithms in providing diagnosis from medical imaging. PLoS One 2020;15:e0238908. 10.1371/journal.pone.0238908 32915901 PMC7485764

[14] Sperrin M Riley RD Collins GS Martin GP . Targeted validation: validating clinical prediction models in their intended population and setting. Diagn Progn Res 2022;6:24. 10.1186/s41512-022-00136-8 36550534 PMC9773429

[15] Riley RD Ensor J Snell KIE . External validation of clinical prediction models using big datasets from e-health records or IPD meta-analysis: opportunities and challenges. BMJ 2016;353:i3140. 10.1136/bmj.i3140 27334381 PMC4916924

[16] Van Calster B Steyerberg EW Wynants L van Smeden M . There is no such thing as a validated prediction model. BMC Med 2023;21:70. 10.1186/s12916-023-02779-w 36829188 PMC9951847

[17] Collins GS Reitsma JB Altman DG Moons KGM . Transparent reporting of a multivariable prediction model for individual prognosis or diagnosis (TRIPOD): the TRIPOD statement. BMJ 2015;350:g7594. 10.1136/bmj.g7594 25569120

[18] Moons KGM Altman DG Reitsma JB . Transparent Reporting of a multivariable prediction model for Individual Prognosis or Diagnosis (TRIPOD): explanation and elaboration. Ann Intern Med 2015;162:W1-73. 10.7326/M14-0698 25560730

[19] Toll DB Janssen KJ Vergouwe Y Moons KG . Validation, updating and impact of clinical prediction rules: a review. J Clin Epidemiol 2008;61:1085-94. 10.1016/j.jclinepi.2008.04.008 19208371

[20] Riley RD Ensor J Snell KIE . Calculating the sample size required for developing a clinical prediction model. BMJ 2020;368:m441. 10.1136/bmj.m441 32188600

[21] Riley RD Snell KI Ensor J . Minimum sample size for developing a multivariable prediction model: PART II - binary and time-to-event outcomes. Stat Med 2019;38:1276-96. 10.1002/sim.7992 30357870 PMC6519266

[22] Shakur H Roberts I Bautista R CRASH-2 trial collaborators . Effects of tranexamic acid on death, vascular occlusive events, and blood transfusion in trauma patients with significant haemorrhage (CRASH-2): a randomised, placebo-controlled trial. Lancet 2010;376:23-32. 10.1016/S0140-6736(10)60835-5 20554319

[23] Steyerberg EW . Validation in prediction research: the waste by data splitting. J Clin Epidemiol 2018;103:131-3. 10.1016/j.jclinepi.2018.07.010 30063954

[24] Riley RD Snell KIE Ensor J . Minimum sample size for developing a multivariable prediction model: Part I - Continuous outcomes. Stat Med 2019;38:1262-75. 10.1002/sim.7993 30347470

[25] van Smeden M de Groot JA Moons KG . No rationale for 1 variable per 10 events criterion for binary logistic regression analysis. BMC Med Res Methodol 2016;16:163. 10.1186/s12874-016-0267-3 27881078 PMC5122171

[26] van Smeden M Moons KG de Groot JA . Sample size for binary logistic prediction models: Beyond events per variable criteria. Stat Methods Med Res 2019;28:2455-74. 10.1177/0962280218784726 29966490 PMC6710621

[27] Riley RD Collins GS Ensor J . Minimum sample size calculations for external validation of a clinical prediction model with a time-to-event outcome. Stat Med 2022;41:1280-95. 10.1002/sim.9275 34915593

[28] Snell KIE Archer L Ensor J . External validation of clinical prediction models: simulation-based sample size calculations were more reliable than rules-of-thumb. J Clin Epidemiol 2021;135:79-89. 10.1016/j.jclinepi.2021.02.011 33596458 PMC8352630

[29] Archer L Snell KIE Ensor J Hudda MT Collins GS Riley RD . Minimum sample size for external validation of a clinical prediction model with a continuous outcome. Stat Med 2021;40:133-46. 10.1002/sim.8766 33150684

[30] Steyerberg EW Harrell FE Jr Borsboom GJJM Eijkemans MJC Vergouwe Y Habbema JDF . Internal validation of predictive models: efficiency of some procedures for logistic regression analysis. J Clin Epidemiol 2001;54:774-81. 10.1016/S0895-4356(01)00341-9 11470385

[31] Riley RD, Collins GS. Stability of clinical prediction models developed using statistical or machine learning methods [Internet]. arXiv; 2022 [cited 2023 Jan 4]. Available from: https://arxiv.org/abs/2211.01061 10.1002/bimj.202200302 PMC10952221 37466257

[32] Harrell FE Jr Lee KL Mark DB . Multivariable prognostic models: issues in developing models, evaluating assumptions and adequacy, and measuring and reducing errors. Stat Med 1996;15:361-87. 10.1002/(SICI)1097-0258(19960229)15:4<361::AID-SIM168>3.0.CO;2-4 8668867

[33] Martin GP Riley RD Collins GS Sperrin M . Developing clinical prediction models when adhering to minimum sample size recommendations: The importance of quantifying bootstrap variability in tuning parameters and predictive performance. Stat Methods Med Res 2021;30:2545-61. 10.1177/09622802211046388 34623193 PMC8649413

[34] Steyerberg EW Bleeker SE Moll HA Grobbee DE Moons KGM . Internal and external validation of predictive models: a simulation study of bias and precision in small samples. J Clin Epidemiol 2003;56:441-7. 10.1016/S0895-4356(03)00047-7 12812818

[35] Steyerberg EW . Clinical prediction models: a practical approach to development, validation, and updating. 2nd ed. Springer, 2019 10.1007/978-3-030-16399-0.

[36] Harrell FE Jr . Regression modeling strategies: with applications to linear models, logistic and ordinal regression, and survival analysis. 2nd ed. Springer, 2015 10.1007/978-3-319-19425-7.

[37] Austin PC van Klaveren D Vergouwe Y Nieboer D Lee DS Steyerberg EW . Geographic and temporal validity of prediction models: different approaches were useful to examine model performance. J Clin Epidemiol 2016;79:76-85. 10.1016/j.jclinepi.2016.05.007 27262237 PMC5708595

[38] Hickey GL Grant SW Murphy GJ . Dynamic trends in cardiac surgery: why the logistic EuroSCORE is no longer suitable for contemporary cardiac surgery and implications for future risk models. Eur J Cardiothorac Surg 2013;43:1146-52. 10.1093/ejcts/ezs584 23152436 PMC3655624

[39] Jenkins DA. Continual updating and monitoring of clinical prediction models: time for dynamic prediction systems? 2021;7. 10.1186/s41512-020-00090-3 PMC7797885 33431065

[40] Booth S Riley RD Ensor J Lambert PC Rutherford MJ . Temporal recalibration for improving prognostic model development and risk predictions in settings where survival is improving over time. Int J Epidemiol 2020;49:1316-25. 10.1093/ije/dyaa030 32243524 PMC7750972

[41] Hippisley-Cox J Coupland C Vinogradova Y . Predicting cardiovascular risk in England and Wales: prospective derivation and validation of QRISK2. BMJ 2008;336:1475-82. 10.1136/bmj.39609.449676.25 18573856 PMC2440904

[42] Riley R Tierney J Stewart L , eds. Individual participant data meta-analysis: a handbook for healthcare research. Wiley, 2021 10.1002/9781119333784.

[43] Steyerberg EW Harrell FE Jr . Prediction models need appropriate internal, internal-external, and external validation. J Clin Epidemiol 2016;69:245-7. 10.1016/j.jclinepi.2015.04.005 25981519 PMC5578404

[44] Royston P Parmar MKB Sylvester R . Construction and validation of a prognostic model across several studies, with an application in superficial bladder cancer. Stat Med 2004;23:907-26. 10.1002/sim.1691 15027080

[45] Takada T Nijman S Denaxas S . Internal-external cross-validation helped to evaluate the generalizability of prediction models in large clustered datasets. J Clin Epidemiol 2021;137:83-91. 10.1016/j.jclinepi.2021.03.025 33836256

[46] Debray TP Moons KG Ahmed I Koffijberg H Riley RD . A framework for developing, implementing, and evaluating clinical prediction models in an individual participant data meta-analysis. Stat Med 2013;32:3158-80. 10.1002/sim.5732 23307585

[47] Debray TPA Collins GS Riley RD . Transparent reporting of multivariable prediction models developed or validated using clustered data: TRIPOD-Cluster checklist. BMJ 2023;380:e071018. 10.1136/bmj-2022-071018 36750242 PMC9903175

[48] Debray TPA Collins GS Riley RD . Transparent reporting of multivariable prediction models developed or validated using clustered data (TRIPOD-Cluster): explanation and elaboration. BMJ 2023;380:e071058. 10.1136/bmj-2022-071058 36750236 PMC9903176

[49] Futoma J Simons M Panch T Doshi-Velez F Celi LA . The myth of generalisability in clinical research and machine learning in health care. Lancet Digit Health 2020;2:e489-92. 10.1016/S2589-7500(20)30186-2 32864600 PMC7444947

[50] Collins GS Altman DG . Predicting the 10 year risk of cardiovascular disease in the United Kingdom: independent and external validation of an updated version of QRISK2. BMJ 2012;344:e4181. 10.1136/bmj.e4181 22723603 PMC3380799

[51] Nevin L PLOS Medicine Editors . Advancing the beneficial use of machine learning in health care and medicine: Toward a community understanding. PLoS Med 2018;15:e1002708. 10.1371/journal.pmed.1002708 30500811 PMC6267950

[52] Collins GS de Groot JA Dutton S . External validation of multivariable prediction models: a systematic review of methodological conduct and reporting. BMC Med Res Methodol 2014;14:40. 10.1186/1471-2288-14-40 24645774 PMC3999945

[53] Moons KGM Altman DG Vergouwe Y Royston P . Prognosis and prognostic research: application and impact of prognostic models in clinical practice. BMJ 2009;338:b606. 10.1136/bmj.b606 19502216

[54] Debray TPA Vergouwe Y Koffijberg H Nieboer D Steyerberg EW Moons KGM . A new framework to enhance the interpretation of external validation studies of clinical prediction models. J Clin Epidemiol 2015;68:279-89. 10.1016/j.jclinepi.2014.06.018 25179855

[55] Cabitza F Campagner A Soares F . The importance of being external. methodological insights for the external validation of machine learning models in medicine. Comput Methods Programs Biomed 2021;208:106288. 10.1016/j.cmpb.2021.106288 34352688

[56] Altman DG Vergouwe Y Royston P Moons KGM . Prognosis and prognostic research: validating a prognostic model. BMJ 2009;338:b605. 10.1136/bmj.b605 19477892

[57] Davis SE Lasko TA Chen G Siew ED Matheny ME . Calibration drift in regression and machine learning models for acute kidney injury. J Am Med Inform Assoc 2017;24:1052-61. 10.1093/jamia/ocx030 28379439 PMC6080675

[58] Wessler BS Nelson J Park JG . External Validations of Cardiovascular Clinical Prediction Models: A Large-Scale Review of the Literature. Circ Cardiovasc Qual Outcomes 2021;14:e007858. 10.1161/CIRCOUTCOMES.121.007858 34340529 PMC8366535

[59] Van Calster B Wynants L Timmerman D Steyerberg EW Collins GS . Predictive analytics in health care: how can we know it works? J Am Med Inform Assoc 2019;26:1651-4. 10.1093/jamia/ocz130 31373357 PMC6857503

[60] Park Y Hu J Singh M . Comparison of Methods to Reduce Bias From Clinical Prediction Models of Postpartum Depression. JAMA Netw Open 2021;4:e213909. 10.1001/jamanetworkopen.2021.3909 33856478 PMC8050742

[61] Ganapathi S Palmer J Alderman JE . Tackling bias in AI health datasets through the STANDING Together initiative. Nat Med 2022;28:2232-3. 10.1038/s41591-022-01987-w 36163296

[62] Vyas DA Eisenstein LG Jones DS . Hidden in Plain Sight - Reconsidering the Use of Race Correction in Clinical Algorithms. N Engl J Med 2020;383:874-82. 10.1056/NEJMms2004740 32853499

[63] Johnson-Mann CN Loftus TJ Bihorac A . Equity and Artificial Intelligence in Surgical Care. JAMA Surg 2021;156:509-10. 10.1001/jamasurg.2020.7208 33625504 PMC8273554

[64] Paulus JK Kent DM . Predictably unequal: understanding and addressing concerns that algorithmic clinical prediction may increase health disparities. NPJ Digit Med 2020;3:99. 10.1038/s41746-020-0304-9 32821854 PMC7393367

