# Supplementary material for: Evaluation of clinical prediction models (part 1): from development to external validation
Source: BMJ. 2024 Jan 8;384:e074819. doi: 10.1136/bmj-2023-074819 (PMC10772854; doi:10.1136/bmj-2023-074819)
Supplement: Supplementary file 1 — Web appendix: Supplementary material [file colg074819.ww.pdf]

SUPPLEMENTARY MATERIAL

Apparent and internal validation of the CRASH-2 model

Supplementary Table 1: Summary measures (mean, standard deviation, and mean percentage bias*) of the apparent and internal (split-sample and bootstrap) performance (c-statistic) of the model to predict in-hospital mortality within 28 days of trauma injury with increasing sample size of the model development study*

		Sample size (for model development)						
		**200**	**300**	**400**	**500**	**1000**	**5000**	**10000**
All data (apparent performance)	Mean	0.861	0.845	0.838	0.833	0.824	0.816	0.816
	Standard deviation	0.037	0.031	0.029	0.024	0.018	0.008	0.005
	Mean percent bias	5.70	3.73	2.85	2.25	1.12	0.016	0.092
Bootstrap correction	Mean	0.786	0.790	0.794	0.797	0.804	0.812	0.814
	Standard deviation	0.050	0.039	0.035	0.029	0.020	0.008	0.005
	Mean percent bias	-3.55	-3.05	-2.55	-2.25	-1.31	-0.365	-0.180
Split sample validation (30%)	Mean	0.745	0.767	0.776	0.783	0.799	0.810	0.812
	Standard deviation	0.096	0.077	0.063	0.056	0.039	0.017	0.011
	Mean percent bias	-8.53	-5.90	-4.79	-3.96	-2.02	-0.573	-0.320

* Percentage bias, which is the relative magnitude of the raw bias to the large sample value (c-statistic = 0.815)

Varying the split ratio

Supplementary Figure 1 shows that regardless of the ratio used to split the available data, there is considerable uncertainty in the performance of the model when evaluated in the test set. Clearly when fewer participants (e.g., 90:10 split) are assigned to the test set, the more variable the model's observed test set performance. For example, in the case study predicting in-hospital mortality within 28 days of trauma injury, at sample size n=200, splitting the data so that 90% are used for model development and 10% to evaluate performance, the observed test set performance in the c-statistic ranges from roughly 0.4 to 1.0.

Supplementary Figure 1: The impact of different split ratios on test performance varying the available sample size when developing and evaluating a model to predict in-hospital mortality within 28 days of trauma injury

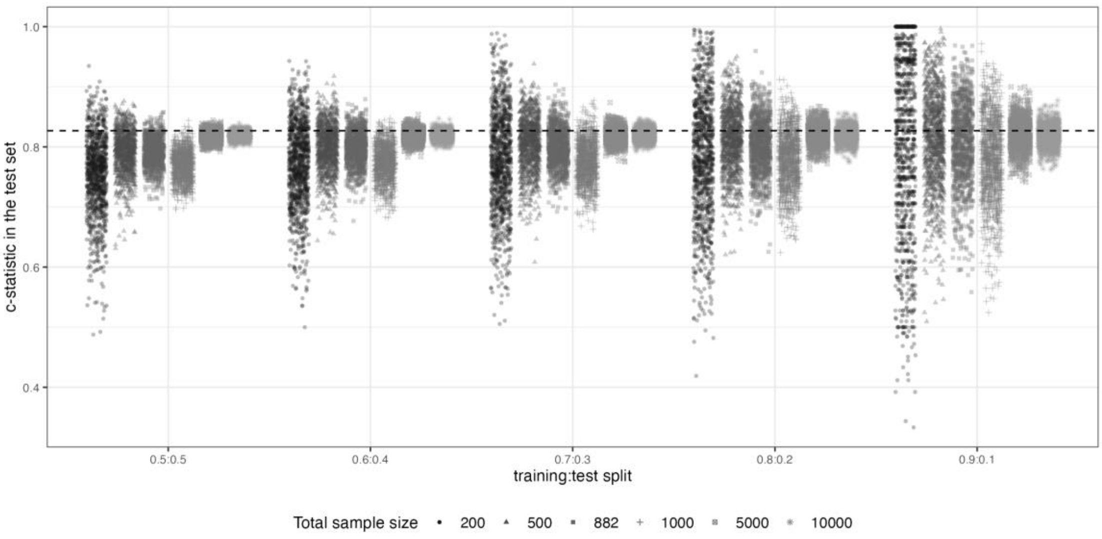

* The dashed black line denotes the large sample c-statistic

External validation of the CRASH-2 Model in CRASH-3 data

Supplementary Figure 2 shows the impact of sample size (and using all the data versus random split sample for model development) on the subsequent performance (an external validation) of the model in new data. For the split-sample approaches, 70% and 50% of the available are being used for model development. The data used to externally validate the model was the CRASH-3 data (n=12743; 2560 deaths)[1]. We assess performance in terms of the c-statistic and the calibration slope (the agreement between the observed and predicted risks across the range of predicted values). When models are developed using small datasets, the subsequent performance upon external validation is poor. For example, the calibration slope mainly has values less than 1, suggesting that predicted risks are too extreme (i.e., too high for individuals at high risk and too low for individuals at low risk). Similarly, the c-statistic is, on average, much lower for models developed using small sample sizes. Moreover, the spread in performance for both performance measures is considerable and thus imprecise, where the c-statistic varies (for example) from 0.592 to 0.805 (lower quartile=0.655, upper quartile=0.790, at n=200, using all the data to develop the model) and 0.520 to 0.798 (lower quartile=0.619, upper quartile=0.785 at n=140, using split-sample approach, i.e., 70% of 200 to develop the model). Model performance at external

validation was systematically better (on average) when all the available data (at the moment of model development) were used to fit the model.

Supplementary Figure 2: External validation of the model using all the data or a split sample (70% and 50%) approach to predict in-hospital mortality within 28 days of trauma injury with increasing sample size of the model development study*

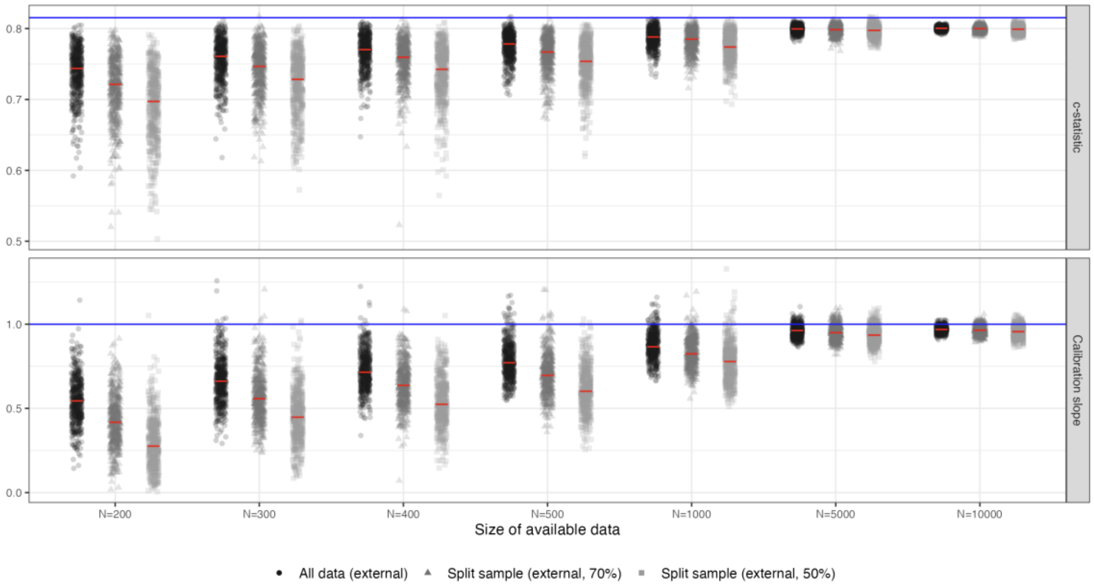

* The blue line for the c-statistic is the performance of the model developed using all the CRASH-2 data (n=20207; 3089 deaths) and evaluated in the CRASH-3 data (n=12743; 2560 deaths), whilst for calibration, the blue line is of perfect calibration (calibration slope=1).

Supplementary Table 2: Summary performance measures for the external validation of the model using all the data or a split sample (70% and 50%) approach to predict in-hospital mortality within 28 days of trauma injury with increasing sample size of the model development study*

		**Sample size (for model development)**						
		**200**	**300**	**400**	**500**	**1000**	**5000**	**10000**
All data	Mean	0.739	0.755	0.767	0.774	0.787	0.799	0.800
	Standard deviation	0.036	0.029	0.024	0.020	0.014	0.005	0.003
	Minimum	0.603	0.622	0.661	0.709	0.735	0.782	0.790
	Maximum	0.805	0.816	0.813	0.814	0.817	0.813	0.807
	Lower quartile	0.650	0.688	0.715	0.727	0.756	0.788	0.794
	Upper quartile	0.792	0.799	0.7804	0.805	0.810	0.808	0.805

Split sample (70% for model development	Mean	0.719	0.740	0.754	0.764	0.782	0.797	0.799
	Standard deviation	0.042	0.035	0.030	0.026	0.016	0.007	0.004
	Minimum	0.552	0.616	0.517	0.671	0.716	0.767	0.788
	Maximum	0.799	0.809	0.809	0.814	0.809	0.813	0.809
	Lower quartile	0.628	0.663	0.688	0.704	0.746	0.784	0.791
	Upper quartile	0.784	0.793	0.798	0.801	0.805	0.809	0.807
Split sample (50% for model development	Mean	0.687	0.717	0.737	0.748	0.772	0.796	0.798
	Standard deviation	0.055	0.043	0.038	0.031	0.020	0.008	0.005
	Minimum	0.508	0.527	0.575	0.624	0.681	0.766	0.782
	Maximum	0.794	0.804	0.808	0.807	0.812	0.815	0.813
	Lower quartile	0.558	0.624	0.647	0.670	0.727	0.779	0.788
	Upper quartile	0.774	0.782	0.793	0.797	0.804	0.810	0.808

Supplementary References

[1] The CRASH-3 trial collaborators. Efficiency of tranexamic acid on death, disability, vascular occlusive events and other morbidities in patients with acute traumatic brain injury (CRASH-3): a randomised, placebo-controlled trial. *Lancet* 2019; 394: 1713—1723.
